# Gene Therapy of Chronic Limb-Threatening Ischemia: Vascular Medical Perspectives

**DOI:** 10.3390/jcm11051282

**Published:** 2022-02-26

**Authors:** Florian Simon, Mansur Duran, Waseem Garabet, Hubert Schelzig, Michael Jacobs, Alexander Gombert

**Affiliations:** 1 Department of Vascular and Endovascular Surgery, Heinrich-Heine-University Düsseldorf, 40225 Düsseldorf, Germany; waseem.garabet@med.uni-duesseldorf.de (W.G.); hubert.schelzig@med.uni-duesseldorf.de (H.S.); 2 Department of Vascular and Endovascular Surgery, Marienhospital Gelsenkirchen, 45886 Gelsenkirchen, Germany; m.duran@marienhospital.eu; 3 Department of Vascular Surgery, University Hospital RWTH Aachen, 52074 Aachen, Germany; mjacobs@ukaachen.de (M.J.); agombert@ukaachen.de (A.G.)

**Keywords:** gene therapy, peripheral arterial disease (PAD), chronic limb-threatening ischemia (CLTI), transduction, transfection, angiogenesis, arteriogenesis, vasculogenesis

## Abstract

A decade ago, gene therapy seemed to be a promising approach for the treatment of chronic limb-threatening ischemia, providing new perspectives for patients without conventional, open or endovascular therapeutic options by potentially enabling neo-angiogenesis. Yet, until now, the results have been far from a safe and routine clinical application. In general, there are two approaches for inserting exogenous genes in a host genome: transduction and transfection. In case of transduction, viral vectors are used to introduce genes into cells, and depending on the selected strain of the virus, a transient or stable duration of protein production can be achieved. In contrast, the transfection of DNA is transmitted by chemical or physical processes such as lipofection, electro- or sonoporation. Relevant risks of gene therapy may be an increasing neo-vascularization in undesired tissue. The risks of malignant transformation and inflammation are the potential drawbacks. Additionally, atherosclerotic plaques can be destabilized by the increased angiogenesis, leading to arterial thrombosis. Clinical trials from pilot studies to Phase II and III studies on angiogenic gene therapy show mainly a mixed picture of positive and negative final results; thus, the role of gene therapy in vascular occlusive disease remains unclear.

## 1. Introduction

Peripheral artery disease (PAD) is one of the three most common manifestations of atherosclerosis and is mainly characterized by intermittent claudication and fatigue, affecting approximately 230 million people worldwide [1]. The most advanced stage of PAD is critical limb ischemia (CLI). It has a poor prognosis with a high probability of limb amputation with limb revascularization (open surgery or endovascular treatment) as the primary treatment option. Despite advances in revascularization methods, many CLI patients are still considered unsuitable for these operations, among other things due to previous operations or general inoperability, and are treated with conservative limb therapies, e.g., best medical treatment of risk factors combined with pain medication and wound treatment [2]. Gene therapy for the treatment of chronic limb-threatening ischemia (CLTI), one of the biological re-vascularization options, could provide new perspectives for those patients without conventional, open or endovascular therapeutic options. The discovery of the proliferative effect of the signaling molecule VEGF (vascular endothelial growth factor) on endothelial cells [3,4,5] marks a milestone in the protein-based, pro-angiogenic therapy for CLTI. The dream of potential success of this approach, the growing interest in the scientific community, as well as the history of failure and drawbacks of these new ideas and therapeutic possibilities is manifested by the increasing number of publications on this subject from the low three-digit range worldwide to about a thousand papers per year. In 2000, PubMed registered 2057 publications under the heading “Angiogenesis”, a number that increased over the years and remains stable between 7000 and 8000 publications per year. However, it should be noted that no distinction has been made between neo-vascularization in vascular disease or other disciplines and as a potential component in tumor biology to cover all aspects of vascular biology. Moreover, in cancer and atherosclerosis, inflammation can cause unregulated angiogenesis, leading to excessive neovascularization, which exacerbates disease [6]. Angiogenesis is an undesired component in the course of tumor development and progression and is therefore the subject of research approaches that will prevent tumor-related angiogenesis; thus, it may be opposed to therapeutic targeting in treatment of CLTI [7].

One problem with biological revascularization is the multifactorial nature of vascular disease, as the following paragraph will show. Both circulating and local mediators of angiogenesis, with signal cascades from the endothelium up to the extracellular matrix, result in such a complex, redundant interaction that it is probably not possible to achieve an effective therapeutic success by using single target therapy. Chronically damaged endothelium leads to a dysfunction and thus has influence on the tissue reactivity. Every single known risk factor, such as hypertension, dyslipidemia, diabetes mellitus, smoking, lack of exercise and age contributes to endothelial dysfunction, so that the vascular biology cannot respond appropriately to new forms of therapy [8,9,10,11,12,13,14,15,16]. However, for angiogenic stimulation, the intact endothelial function is of crucial importance. The reduced release of nitric oxide (NO) by the endothelium is a key factor in this context, which is reduced in chronic injury. Endothelial cells, which proliferate at the moment, emit a multiple of NO as compared to quiescent cells [17]. The inhibition of NO by the antagonist L-NG-nitroarginine-methyl-ester (L-NAME) leads to a reduced response of the endothelium towards stimulation by the factor VEGF in combination with a decrease in the NO concentration [18]. A number of other studies show the significant relationship between NO production and angio- and arteriogenesis [19,20,21,22,23,24]. Another example of interfering factors are free radicals formed in ischemic tissues. They also play a critical role in diabetes [25,26,27,28], in which they contribute to the oxidation of structural proteins and cause, by the conversion of NO to peroxynitrite (ONOO^−^), the consumption of NO [29,30,31].

Thus, a therapeutic success depends at the end on both the right agent, as well as the condition and reactivity of the target tissue itself [13,14,22,32,33].

## 2. Gene Therapy Techniques: Transduction vs. Transfection

The principle underlying gene-based therapy is to introduce genes or genome segments into cells. There are two approaches for delivery: transduction and transfection.

### 2.1. Viral Transduction

**Figure 1:**
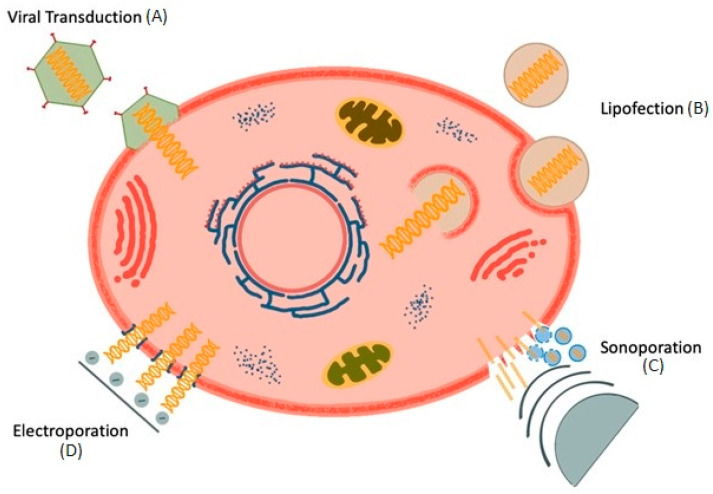
Gene therapy techniques. (**A**) Transduction uses a vector to deliver genetic material into the cell. (**B**) Lipofection: Liposomes become incorporated by the cell and releases genetic material after destruction of the endosome membrane (**C**) Sonoporation: Genetic information gets channeled either directly by ultrasound or via bursting microbubbles (**D**) Electroporation brings genetic material via electrically conductive pores into the cell.

In the case of transduction, viral vectors are used, in which the original DNA is replaced or additional genes are introduced into the viral genome [34,35,36,37]. The prepared virus binds to the target cell, the nucleocapsid is introduced into the cytoplasm, and the sheath then removed (Figure 1). The released viral genome remains within the cytoplasm or is shuttled into the nucleus. Depending on the strain of the virus used, a transient or long-lasting signaling can be achieved. With, for example, adeno-associated virus (AAV) transduction, the new genetic segments are permanently integrated into the host genome, where it can induce the production of proteins. On the one hand, AAVs carry only a small genome through which only relatively small amounts of information can be transported. On the other hand, AAVs appear to be of low pathogenicity and can achieve long-lasting effects through permanent expression of their genetic material. In contrast, although adenoviruses can transport a larger amount of genetic information and also integrate into a wide range of different cells, they can usually only cause short-term effects due to their episome and often bring about pronounced immune reactions [38]. Furthermore, it is possible that the introduced DNA or RNA encodes therapeutic molecules and that the production of proteins is induced directly in the cell itself, such as the aforementioned VEGF [39,40,41,42,43,44].

The theory behind this approach sounds relatively simple, as well as plausible. In the clinical context, adeno-associated virus (AAV)-based trials are a therapeutic option of ever-increasing importance. The strength this vector-based therapy is also its weakness. As AAVs are part of the natural environment and infections are widespread, many patients have pre-existing antibodies against AAVs [37]. AAV therapy itself can also trigger the formation of antibodies [45]. The individual expression of the immune response varies depending on pre-existing antibodies, the serotype of the AAV, the age of the patient, etc. These antibodies can have a varying influence on the effectiveness of a vector-based therapy through the interception of the AVV by the antibodies.

The interactions range from the uptake of the vector into the tissue to intracellular processes, such as the transfer of genetic material into the cell nucleus [46]. It is unclear whether preformed anti-AAV antibodies can be considered a prognostic factor for the success of a therapy, and if so, at what titre. The presence of a threshold level of pre-existing antibodies reduces the transduction of i.v.-administered AAV vectors. Initial studies at least indicate that titres of less than 1:400 have no influence on the transduction of an AVV. High levels of pre-existing antibodies may also promote phagocytosis, preventing transgene expression. The suppression of innate immunity may therefore have positive effects on the transduction efficiency in individuals with high antibody titres [47,48]. Fears of an off-target delivery of the vector and the associated expression of proteins in other organs have been questioned but denied employing adenovirus or foamy virus vectors [44,49,50,51].

Foamy viruses are non-pathogenic retroviruses, usually found in mammalian animals, with a large tissue tropism. Replication-defective foamy virus vectors (FVVs) were shown to transfer marker genes efficiently into repopulating mouse and ex vivo human cells and have proved to be safe in in vivo experiments with non-human primates [44]. The risk of malignant transformation when inserted into the genome (e.g., in case of retroviral transductions) or uncontrolled gene expression, as well as inflammatory responses to viral epitopes, has not been eliminated [52,53,54].

### 2.2. Transfection

In contrast to the transduction of DNA by viruses, exogenous DNA is introduced directly into cells, for example, by lipofection, electro- or sonoporation [55,56,57,58,59,60,61].

#### 2.2.1. Lipofection

Examples of chemical transfection are liposomes or polymers [62]. Liposomes consist of a lipid bilayer membrane, which is hydrophilic on the outside and the inside but lipophilic between the two lipid layers. In this way, both fat- and water-soluble substances can be transported by liposomes that, depending on the corresponding solubility, incorporate between the lipid layers or in the core. By the fusion of liposomes with the cell wall of the target cells, the introduced substance in the liposome may be released into the cytosol and act there either directly or, in the case of DNA, initiate the production of corresponding proteins. As a vehicle for the therapeutic genes, plasmids are often used [63,64,65,66,67,68,69,70,71,72,73,74,75,76,77]. Plasmids are circular DNA, existing as extrachromosomal DNA in the cytosol and having a cell-independent replication cycle. If a plasmid is incorporated into the DNA of the host and is thus no longer extrachromosomal, the plasmid is called an episome. These are then no longer replicated autonomously but, as part of the natural DNA, take place in the normal cell cycle. The advantage of this is that episomes cannot replicate themselves uncontrollably.

#### 2.2.2. Electroporation

Electroporation, as another form of transfection, works either with short electric impulses of high voltage or with several pulses of longer duration at a low voltage to polarize the cell membrane to pass the target DNA into the cell interior via electrically conductive pores [78,79,80]. If the applied voltage is high enough, openings can also be created in the lipid double membrane of a cell. By realigning the lipid molecules according to the applied voltage, hydrophilic channels can be created. Factors such as the level of the applied voltage, the shape and radius of the affected cell, the stimulation duration and the ambient temperature, as well as several other variables, are directly related to each other and explain the different response of different cell types. There are several ways of doing this. Firstly, an electrical voltage can be applied to a cell suspension. This creates pores of different sizes distributed over the cell surface, which can take up the most diverse forms of agents, such as proteins or plasmids. Factors such as the type of cell, the size of the pores and the charge of the agent play a decisive role in whether and how much agent can be absorbed into the cell. This type of electroporation is useful for transfecting larger quantities of cells of a certain cell type, but a precise dosage of the intracellularly introduced agent is hard to perform. On the other hand, the field of miniaturized electroporation opens up new perspectives. With this technique, it is possible to transfect individual cells in a targeted manner and thus have better control over the dose and survival of the transfected cells. By passing the cells through a kind of membrane system, larger quantities of cells can be transfected, despite the single electroporation. Since only low voltages are necessary here, more cells survive the procedure than in a general cell suspension. However, there is then still the question of the application of the cells into an organism [80]. This method for the treatment of PAD muscle ischemia has been successfully tested in animal models [81,82,83]. In humans, this technique has, however, only been used experimentally in cancer therapy and vaccine development [58,84,85,86,87].

#### 2.2.3. Sonoporation

Another form of physical transfection of DNA is sonoporation. In this procedure, the plasmid is either channeled by ultrasound directly into the cell [61,88,89,90] or transported to the cell via ultrasound contrast agents dissolved in microbubbles. Heat-denatured human serum albumin microspheres filled with, e.g., perfluorocarbon [91,92,93], containing the DNA serve as a vehicle. Using ultrasound, the bubbles burst and set the genetic information free locally [61,94,95,96]. Possible kinds of application are the systemic administration, with distribution via the arterial residual perfusion and the direct intramuscular injection into the desired target tissue. However, even this technique involves extensive fine-tuning of parameters to ensure the optimal application. If cells are treated with a higher sound intensity, cell damage may result. Although this increases the penetration depth and the absolute amount of agent, this can be at the expense of cell function. The increase in permeability comes at the cost of increased cell destruction. However, not only does the direct effect of the sound waves on the cells leads to cell destruction but so do the microbubbles themselves. The closer the transport bubbles are to the cell membrane, the greater the damaging effect on the cells. The bursting of the microbubbles can also lead to mechanical damage due to the immediate proximity to the cell wall. Although this again increases the penetration depth of the agent, the cell damage also increases. Finally, the duration of the treatment has a significant influence on both the available intracellular amount of the agent and the cell damage. A shorter pulse duration protects the cell structures and also increases the penetrance of the agent [97]. To obtain the best possible results, the optimal distance between bubbles and cell wall should be defined in combination with the acoustic pressure. Future clinical studies will then have to show to what extent such complicated tunings can be implemented in reality.

## 3. Risks Associated with Gene Therapy

There are disadvantages and side effects associated with gene therapy [98,99]. The emphasis here is that increasing angiogenesis causes vessel growth not only in desired tissues. There is a risk that such processes become autonomous. In the course of a systemic reaction, vessel growth could then be held in unwanted tissues. The formation of vascular-based diseases or promoting hitherto dormant pathogenic processes could be set in motion. A key factor in malignant tumor progression is the resulting hypoxia in the expanding tissue and the need for an adequate supply of the neoplasm with blood. The tumor induces angiogenesis using signal molecules such as, e.g., HIF-1α, VEGF, FGF, HGF [100,101,102,103]. The vessels arising by this cytokine’s stimulation are, in contrast to the desired therapeutic angiogenesis in PAD therapy, messy and can ensure the blood supply of the tumor only marginally, which ends in renewed hypoxia of the neoplasia [104,105,106]. If a gene-based therapy for the treatment of PAD would meet an occult or an already manifest tumor, the possible tumor progression could have fatal consequences for the patient. Angiogenesis of the extremities induced by gene therapy can be the reason that tumor tissue benefits from this. On the one hand, current therapies may be adversely affected or diminished; on the other hand, occult tumors could get into progression.

For example, a disease which can accelerate with increased angiogenesis is proliferative retinopathy [107]. Proliferative retinopathy is a concomitant illness observed with both types of diabetes mellitus. The reason for this is that microangiopathy of the retina leads to an insufficient supply of blood in the eye and therefore causes increasing angiogenesis, as a natural response to hypoxic stress. In combination with an angiogenic therapy of the PAD, an additive process can take place, which leads at the end to blindness. However, there are also dangers for patients who are supposed to benefit from gene therapy. Atherosclerotic plaques, as present in PAD, can be destabilized by increased angiogenesis, break up and cause the onset of a clotting response and thus lead to arterial thrombosis [108,109,110,111,112]. Additionally, in treating chronic peripheral arterial disease, an acute limb ischemia caused, e.g., by arterial embolism can occur, which is associated with increased morbidity and mortality. According to current guidelines, these concerns have not occurred in clinical trials [113].

## 4. Clinical Trials Based on Gene Therapy and Perspective

Clinical trials from pilot studies to Phase II and III studies on angiogenic gene therapy show mainly a mixed picture of positive and negative final results. In these studies, growth factors such as VEGF [39,40,41,43,62,63,64,65,66,68,70,71], FGF [67,75,76,114,115,116,117] and HGF (hepatocyte growth factor) [69,73,74,118,119] were examined, and both viruses and plasmids were employed as delivery vehicles. Most therapeutic agents have been applied directly via intramuscular injection, as balloon surface coating or as an intra-arterial infusion via the femoral artery. The primary endpoints such as improvement of vascularization, walking distance, rate of amputation, ulcer healing, percutaneously measured oxygen, partial pressure and pain at rest showed both positive and negative effects. So far, no unified picture has emerged showing the fundamental success of gene therapy [120,121,122].

Up to now, applied gene therapy has had no safety-relevant side effects [122,123]. In 2008, the TALISMAN trial included 125 patients and reported a significant improvement in Amputation-free survival at 12 months in 73% of patients treated with FGF plasmid compared with 48% in placebo-treated patients with no options for revascularization [75]. Studies published in 2009 suggested a potential beneficial usage of gene therapy in CLTI patients: The application of riferminogen pecaplasmid (NV1FGF) promotes local angiogenesis by stimulating cell migration and cell growth and appears to induce the formation of new blood vessel networks [117]. The results from a Phase III study (TAMARIS) involving 525 patients with critical limb ischemia showed no significant differences between the investigated drug, NV1-FGF, and the placebo group with respect to the primary endpoints (time to major amputation or death after one year) and the secondary endpoints (e.g., minor amputations, skin lesions, pain intensity and ankle–brachial index) [76].

A study published in 2018 looked at the effects of therapy with a plasmid (pl-VEGF165) in a 5-year follow-up. This study assessed the long-term safety of the drug and the efficacy of angiogenesis induction in 36 patients with atherosclerosis-related chronic lower limb ischemia compared to 12 control patients. The solution was administered as 5–10 intramuscular injections into the calf muscles twice 1.2 mg at 14-day intervals (a total dose of 2.4 mg). It was shown that with regard to the safety of the drug, there was no abnormality in terms of cardiovascular events, development of tumor neoplasms or changes in visual acuity in the group comparison. In the therapy group, limb salvage was 95% compared to 67% in the control group. The pain-free walking distance increased from an average of 105 m to 384 m. The ankle–brachial index increased from an average of 0.45 to 0.56 after the first year to 0.51 at the end of the 5-year follow-up. The TcPO_2_ value increased from an average of 66.7 mmHg to 84.1 mmHg after 5 years and was thus not significantly higher than in the control group with 73.6 mmHg. The research group concludes positively that the use of pl-VEGF165 is well tolerated and does not lead to the formation of tumors, cardiovascular complications or impaired vision due to vascular sprouting in the retina. It is also concluded that if pl-VEGF165 is used before necrotic-ulcerative changes occur, the therapeutic effect of pl-VEGF165 will last for at least 5 years [124].

Despite a wide variety of results and setbacks, there is still a lot of hope in the idea of gene therapy. The STOP-PAD study from 2020 shows the unchanged great interest in a gene therapy option. This study presents the effects of treatment with a Stromal Cell-Derived Factor-I plasmid. The study was designed as a multicenter, randomized, double-blinded, placebo-controlled phase 2B study. The purpose of the study was to evaluate the impact on outcome of patients with CLTI at or below the knee level after successful arterial revascularization with persistent circulatory disturbance in the forefoot. The plasmid was administered by intramuscular injection, with one group receiving 8 mg and another group 16 mg of the plasmid and compared to a control group. A total of 109 patients were included. The primary objective was to evaluate wound conditions after 6 months and the incidence of major adverse limb events (MALE). The results showed a regrettably homogeneous distribution pattern of both positive and negative changes across all three groups. After 6 months, only one-third of the patients in each group showed improved wound conditions. Although there were statistically significant improvements in the toe–brachial index, these positive changes applied equally to all three groups but ultimately did not lead to a reduction in MALE. Ultimately, this study could not show an improvement in outcome, although the combination of revascularization with postoperative gene therapy using a non-viral DNA-based plasmid seemed promising [125].

So far, the current guidelines of the European Society of Vascular Surgery (ESVS) do not mention gene therapy as clinical applicable option for the treatment of CLTI [126]. In 2020, the Global Vascular Guidelines on the Treatment of Peripheral Artery Disease supports the recommendations given in 2018 by the ESVS, as they were not able to give a clear recommendation for gene therapy for patients without interventional therapeutic option [113]. Despite the very mixed results of previous studies, it is worth continuing to keep an eye on gene therapy. Experimental studies show results that give rise to some hope. A research group proposes a novel and easy-to-implement non-viral approach to topical tissue reprogramming, validated with existing and newly developed reprogramming models of induced neurons and endothelium, respectively, via a nanochannel system [80].

## 5. Limitations of Clinical Use of Gene Therapy

There are several reasons why gene therapy is still in its infancy. On the one hand, previously approved gene therapies are designed for rare diseases that are based on a specific and defined factor in the genome. For example, the first gene therapy approved in Europe was Glybera in 2012 for the treatment of the hereditary disease lipoprotein lipase deficiency (LPLD). This disease affects just less than 1000 people in the European Union [127]. Therapies such as Strimvelis and Luxturna followed. Strimvelis is a gene therapy for the rare immune disease ADA-SCID, approved in the EU in 2016, in which the formation of white blood cells is impaired. In patients, previously harvested stem cells are transduced in the laboratory with healthy gene segments using a retrovirus. These manipulated stem cells are then returned to the patient [128]. The treated patients were followed up for a mean of 7 years and showed very good treatment success [129]. However, the cost was EUR 594,000 per treatment.

Luxturna treats early retinal dystrophy using an AAV vector applied directly under the retina. The cost is approximately USD 830,000 per treatment [130]. Other examples of approved gene therapies are Zynteglo for ß-thalassemia (cost of EUR 1.6 million spread over 5 years), Zolgensma for spinal muscular atrophy (EUR 2.2 million) and Libmeldy for metachromatic leukodystrophy (price not yet fixed) [131,132,133].

Although the therapies mentioned are not approved procedures for the treatment of critical leg ischemia, the problems of gene therapy can be well illustrated by the examples given. On the one hand, the development of such therapies is lengthy and very expensive, especially since gene therapies have so far been developed primarily for rare hereditary diseases and thus had to contend with long recruitment phases. The strength of gene therapy for vascular patients therefore lies primarily in the wide spread of the disease compared to rare diseases. At the same time, however, the weakness of vascular-based gene therapy is also evident here: the lack of the clearly assignable gene defect that would have to be remedied.

Another point that complicates gene therapy is the costly production compared to conventional drugs. Highly specialized laboratories produce individualized drugs for patients. However, a general approach, if at all definable, as would be necessary in vascular medicine, could offer an advantage here and reduce treatment costs. From the data reported above on gene therapy in vascular medicine, however, this is precisely the sticking point: There is a lack of effective, broad-based therapy regimens, as it is not possible to define a single gene defect or similar.

## 6. Conclusions

Gene therapy, as described above, was and is rightly the subject of many studies. The idea to make a direct and lasting therapeutic success through the manipulation of genes is impressive. By the use of various techniques, such as transduction and transfection, with various vehicles, such as adenoviruses or plasmids, the success seemed to be within reach. Growth factors such as VEGF, FGF and HGF have been extensively investigated but show heterogeneous effects, both positive and negative, with respect to the primary endpoints such as improvement of vascularization, walking distance, rate of amputation, ulcer healing, transcutaneously measured oxygen partial pressure and pain at rest. The results from a Phase III study (TAMARIS) and a 2B study (STOP-PAD) showed a very good tolerability of the therapy, but were negative in all endpoints, therefore questioning the success of gene therapy. So why could clinical phase II and III studies not confirm the hopes placed in it? Are these disappointing clinical results of gene therapy due solely to a failure of the agent, or is it a much more complex event in the context of atherosclerosis? Technological advances, in combination with other tools such as molecular engineering, will further advance biomedical research in the field of regenerative medicine. The future of gene therapy in vascular medicine depends on the definition of targets that can be addressed by gene therapy in combination with a reduction in treatment costs.

Future clinical studies will then have to prove the feasibility of such molecular high-tech therapeutics in reality.

## Data Availability

Not applicable.
